# Effects of long‐term exercise training for different durations on pancreatic amylase activity and intestinal glucose transporter content in rats

**DOI:** 10.14814/phy2.14255

**Published:** 2019-10-24

**Authors:** Saki Kondo, Ayumi Fukazawa, Takuya Karasawa, Shin Terada

**Affiliations:** ^1^ Department of Life Sciences Graduate School of Arts and Sciences The University of Tokyo Tokyo Japan; ^2^ Research Fellow of Japan Society for the Promotion of Science Tokyo Japan

**Keywords:** Amylase, GLUT2, long‐term exercise training, SGLT1

## Abstract

Long‐term endurance training for a relatively short duration (~1 h) is reported to increase pancreatic amylase activity in rats, suggesting that chronic exercise training enhances carbohydrate digestive capacity. However, it remains unknown whether longer exercise training duration results in greater adaptation in the pancreas and small intestine. Thus, this study aimed to examine the effects of long‐term endurance training for a longer duration on pancreatic amylase activity and intestinal glucose transporter content in rats. Male Sprague–Dawley rats were subjected to swimming exercise training for 1 h (Ex‐1h group) or 6 h (Ex‐6h group, two 3‐h sessions separated by 1 h of rest) each day, 5 days a week, for 6 weeks. Sedentary rats were used as a control (Con group). Total pancreatic amylase activity in the Ex‐6h group was significantly lower than that in the Con and Ex‐1h groups immediately after the last training session. After 24 h of recovery, total pancreatic amylase activity was significantly higher in the Ex‐1h group (~46%) than in the Con group, and a further increase was observed in the Ex‐6h group (~98%). In addition, the Ex‐6h group, but not the Ex‐1h group, showed significantly greater intestinal sodium‐dependent glucose transporter 1 (SGLT1) content compared with the Con group after 24 h of recovery. However, no significant difference was observed in glucose transporter 2 (GLUT2) content among the three groups. In conclusion, chronic endurance exercise training for a longer duration results in larger increases in pancreatic amylase activity and intestinal SGLT1 content in rats.

## Introduction

During prolonged intense exercise (and the most competitive events), carbohydrate (CHO) is the major energy fuel. Fatigue during endurance exercise is often correlated with glycogen depletion in muscles and reduced glucose concentrations in blood (Bergstrom et al. 1967; Coyle et al. 1986; Holloszy et al. 1998). Thus, to improve performance during prolonged intense exercise, intake of CHOs before and during exercise is generally necessary to increase glycogen stores and CHO oxidation in skeletal muscle (Coggan et al. 1991; Jeukendrup 2004).

The gastrointestinal (GI) tract is an important step in the delivery of exogenous CHO to skeletal muscle, and digestive and absorptive capacity in the GI tract can therefore be a major determinant of endurance exercise performance. Because ingested CHOs are digested by pancreatic amylase and then absorbed in the small intestine by sodium‐dependent glucose transporter 1 (SGLT1) and glucose transporter 2 (GLUT2) (Shirazi‐Beechey 1995), the digestive and absorptive capacities for CHO might be determined by pancreatic enzyme activity and intestinal transporter content. A large body of evidence suggests that these GI systems are highly adaptable (Jeukendrup 2017). However, only a few studies have explored the effects of long‐term endurance training on pancreatic amylase activity and intestinal glucose transporter content (Zsinka and Frenkl 1983; Kugino and Kishino 1991; Minato 1997). For instance, Minato (1997) demonstrated that performing 1 h of running exercise per day, 5 days per week, for 6 weeks induced an increase in pancreatic amylase activity and pancreas hypertrophy in rats. These results indicate that long‐term endurance training induces adaptation in the digestive capacity of the pancreas. However, it remains unknown whether the more strenuous endurance exercise training that is usually performed by athletes can induce more substantial adaptation in the pancreas, and whether intestinal glucose transporter content can also adapt to long‐term endurance exercise training. In addition, although long‐term endurance exercise training increased total pancreatic protein content (Minato 1997), which reflects digestive enzyme content including amylase (Logsdon and Ji 2013), the effects of endurance training on the protein synthesis and degradation (e.g., autophagy) pathways in pancreas have been less clear.

Against this background, this study examined the effect of long‐term endurance exercise training for different durations on pancreatic amylase activity, total pancreatic protein content, and intestinal glucose transporter content in rats and clarify the mechanism by which endurance exercise training induces the adaptations in rat pancreas.

## Materials and Methods

### Animals and exercise protocol

Six‐week‐old male Sprague–Dawley (SD) rats (CLEA Japan, Tokyo, Japan) with body weights of 130–150 g were kept in individual cages. The environment was maintained at 22 ± 1°C, with 50 ± 5% humidity with lights turned on from 09:00 to 21:00. All animals were treated in accordance with national guidelines for the care and use of laboratory animals (Notification of the Prime Minister’s Office of Japan). The Animal Experimental Committee of The University of Tokyo approved this experimental protocol.

After a 7‐day acclimatization period, the rats were divided into three groups: a control (Con) group, a 1‐h swimming training (Ex‐1h) group, and a 6‐h swimming training (Ex‐6h) group. All animals were acclimated to swimming exercise for 10 min per day on the 3 days before being divided into these groups. Mean body weight and food efficiency during the acclimatization period were matched among the three groups. While rats in the Con group remained sedentary, rats in the Ex‐1h and Ex‐6h groups performed swimming exercise for 1 h (Ex‐1h group) or 6 h (Ex‐6h group, two 3‐h exercise sessions separated by 1 h of rest) each day, 5 days a week. This level of training was maintained for 6 weeks. Rats in both training groups swam in a barrel filled to a depth of 45 cm in groups of 6 or 7. The average surface area was 200–230 cm^2^/rat. The temperature of the water was kept at 35 ± 1°C during the swimming exercise. Previous studies demonstrated that 1 h of exercise each day, 5 days a week, for 6 weeks induced an increase in pancreatic amylase activity and pancreas hypertrophy in rats (Minato 1997), and 6‐h swimming exercise training has been considered the strongest stimulus for inducing biochemical adaptations in rat skeletal muscle (Ren et al. 1994; Nakatani et al. 1997; Terada et al. 2001).

All the rats were fed an AIN‐93G‐based diet (Tousen et al. 2016) (composition in g/kg): corn starch 529.486 g, casein 200 g, sucrose 100 g, canola oil 70 g, cellulose powder 50 g, mineral mix (AIN‐93G) 35 g, vitamin mix (AIN‐93) 10 g, l‐cystine 3 g, choline bitartrate 2.5 g, tert‐Butyl hydroquinone 0.014 g. They were allowed free access to food and water, except for 7 h while rats in the Ex‐6h group were exercising. Food intake and body weight were recorded every other day.

Following the last training session, rats in each group were further separated into two groups and were then sacrificed either immediately after or 24 h after the last training session. Rats that were to be sacrificed 24 h after the last training session (after a 24‐h recovery period) were returned to the cage and were allowed free access to food and water.

Rats were sacrificed under anesthesia with isoflurane. Blood samples were collected from the heart and then centrifuged at 4000 × *g* for 10 min. The epitrochlearis muscle, intra‐abdominal fat, and pancreas were removed and weighted. The epitrochlearis muscle and pancreas samples were frozen in liquid N_2_. The jejunum was dissected and washed in 0.9% NaCl, after which its mucosa was scraped with a spatula and stored in a Radio‐Immuno Precipitation Assay (RIPA) lysis buffer (Merck Millipore, Billerica, MA) containing 50 mmol/L Tris‐HCl pH 7.4, 150 mmol/L NaCl, 0.25% deoxycholic acid, 1% NP‐40 and 1 mmol/L Ethylenediaminetetraacetic acid (EDTA) with protease inhibitor cocktail (Sigma‐Aldrich, St. Louis, MO), previously dispensed in a 1.5 mL microtube. The samples were stored at −80°C until analysis.

### Pancreatic amylase activity measurement

A portion of the pancreas was homogenized in an ice‐cold assay buffer and centrifuged at 12000 × *g* for 5 min at 4°C. Amylase activity in the supernatant was analyzed with the Amylase Assay kit (Abcam, Cambridge, UK) according to the manufacturer's instructions.

### Sample homogenization

Another portion of the pancreas, epitrochlearis muscle, and jejunum were homogenized in an ice‐cold RIPA lysis buffer with a protease inhibitor cocktail. Phosphatase inhibitors (PhosSTOP; Roche, Basel, Switzerland) were added to the RIPA lysis buffer for the pancreas samples. The homogenates were subjected to three freezing‐thawing cycles to disrupt intracellular organelles before being rotated end‐over‐end at 4°C for 60 min to solubilize the protein. A bicinchoninic acid (BCA) protein assay kit (Thermo Fisher Scientific, Waltham, MA) was used to measure total pancreas protein content. The homogenized samples were centrifuged at 700 × *g* for 5 min at 4°C after which the supernatants were collected.

### Western blot analysis

A BCA protein assay kit was used to measure the protein concentrations in supernatants of epitrochlearis muscle, pancreas, and jejunum. Samples were prepared in a Laemmli buffer consisting of 0.25 mol/L Tris‐HCl, 0.02% (w/v), Bromophenol Blue (BPB), 8% (w/v) Sodium dodecyl sulfate (SDS), 40% (w/v) Glycerol, and 20% (v/v) 2‐mercaptoethanol, at a pH of 6.8 (FUJIFILM Wako Pure Chemical Corporation, Osaka, Japan). The mixture was heated at 95°C for 5 min in a heating block. The epitrochlearis muscle sample for GLUT4 measurement was prepared without using 2‐mercaptoethanol and boiling. The protein sample was evenly divided and subjected to SDS‐polyacrylamide gel electrophoresis (% of polyacrylamide in resolving gels: 7.5% for SGLT1 and phospho‐p70S6k, 10% for GLUT2 and GLUT4, and 15% for microtubule‐associated protein light chain 3 [LC3]) (Laemmli 1970) and then transferred to polyvinylidene difluoride membranes (Merck Millipore) at 200 mA for 90 min. Following transfer, membranes were blocked for 1 h at room temperature in Tris‐buffered saline (TBS) with 0.1% Tween20 (TBS‐T; 20 mmol/L Tris base, 137 mmol/L NaCl, pH 7.6) supplemented with 5% (w/v) nonfat powdered milk. The membranes were incubated at 4°C overnight with a primary antibody dilution of 1:1000 or 1:10,000 in TBS‐T containing 5% bovine serum albumin using: anti‐GLUT2 (#07‐1402; Merck Millipore), anti‐SGLT1 (#07‐1417; Merck Millipore), anti‐LC3 (#PM036; Medical & Biological Laboratories, Nagoya, Japan), anti‐phospho p70S6k (#9234; Cell Signaling Technology, Danvers, MA) and polyclonal antiserum specific for GLUT4 from the laboratory of Dr. John O. Holloszy (Washington University, St. Louis, MO). The membranes were then incubated at room temperature for 1 h with secondary antibodies (anti‐mouse IgG or goat anti‐rabbit IgG; Jackson ImmunoResearch, West Grove, PA) at a dilution of 1:5000 in TBS‐T containing 1% nonfat powdered milk. Bands visualization was performed using an enhanced chemiluminescence prime reagent (GE Healthcare, Buckinghamshire, UK) and quantified by Image Studio Digits (Ver. 5.2; LI‐COR Biosciences, Lincoln, NE). To verify equal protein loading across lanes, membranes were stained with Ponceau (Sigma‐Aldrich).

### Glycogen measurement

The epitrochlearis muscles were homogenized with 0.3 mol/L perchloric acid. After acid hydrolysis, the glycogen concentration was measured using enzymatic methods described by Lowry and Passonneau (1972).

### Intestinal fatty acid binding protein (I‐FABP) measurement

Serum I‐FABP concentration was measured with Rat intestinal fatty acid binding protein (iFABP) ELISA Kit (MBS265971; MyBioSource, San Diego, CA).

### Statistical analysis

Data are presented as the mean ± standard error of the mean (SEM). One‐way analysis of variance (ANOVA; Social Survey Research Information, Tokyo, Japan) was used to compare differences among the three groups. When ANOVA detected a significant main effect, post hoc analyses were performed using Tukey‐Kramer tests. Associations between variables were determined using Pearson’s correlation coefficient. A value of *P* < 0.05 was defined as the threshold of statistical significance.

## Results

### Final body weight, total food intake, and intra‐abdominal fat weight

Final body weight, total food intake, and intra‐abdominal fat weight are shown in Table 1. In the Ex‐6h group, final body weight was significantly lower than that in the Con and Ex‐1h groups both immediately (vs. Con: *P* < 0.001, vs. Ex‐1h: *P* = 0.008) and 24 h after the last exercise session (vs. Con: *P* < 0.001, vs. Ex‐1h: *P* = 0.002). Total food intake during the 6‐week intervention was not significantly different among the three groups of rats sacrificed either immediately or 24 h following the last exercise session.

**Table 1:** Final body weight, total food intake, and intra‐abdominal fat weight.

	Immediately after exercise			24 h after exercise		
	Con	Ex‐1h	Ex‐6h	Con	Ex‐1h	Ex‐6h
Final body weight (g)	457.7 ± 13.4	411.4 ± 9.5	345.5 ± 14.1^***,††^	457.4 ± 12.6	411.2 ± 9.2^*^	345.1 ± 5.4^***,††^
Total food intake (g)	949.9 ± 31.9	924.6 ± 22.6	962.3 ± 41.5	945.9 ± 26.2	906.1 ± 15.8	970.4 ± 17.0
Intra‐abdominal fat weight (g)	25.3 ± 2.1	14.6 ± 1.6^**^	8.5 ± 0.8^***^	28.6 ± 2.5	14.8 ± 1.8^***^	8.7 ± 1.0^***^

Con, control group; Ex‐1h, 1‐h swimming training group; Ex‐6h, 6‐h swimming training group.

Rats were sacrificed immediately or 24 h after the last exercise session, respectively.

Values are presented as means ± SEM. *n* = 6–8. *P < 0.05, **P < 0.01, ***P < 0.001 vs. Con. ^††^ *P* < 0.01 vs. Ex‐1h.

Intra‐abdominal fat weight in rats sacrificed immediately after the last exercise session was significantly lower in the Ex‐1h and Ex‐6h groups than in the Con group (vs. Ex‐1h: *P* = 0.001, vs. Ex‐6h: *P* < 0.001). Moreover, intra‐abdominal fat weight in the Ex‐1h and Ex‐6h groups were significantly lower compared with that in the Con group after 24‐h recovery (vs. Ex‐1h: *P* < 0.001, vs. Ex‐6h: *P* < 0.001).

### GLUT4 content and glycogen concentration in the epitrochlearis muscle

**Figure 1:**
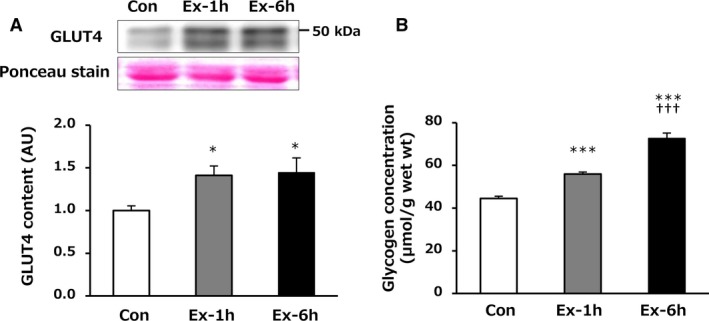
Effects of 6‐week swimming exercise training on GLUT4 content (A) and glycogen concentration (B) in the epitrochlearis muscle after 24‐h recovery. Con, control group; Ex‐1h, 1‐h swimming training group; Ex‐6h, 6‐h swimming training group; AU, arbitrary unit. Values are means ± SEM. *n* = 6–8. *P < 0.05, ***P < 0.001 vs. Con. ^†††^ *P* < 0.001 vs. Ex‐1h.

To confirm whether the endurance exercise training regimen examined in this study elicited adaptations in rats, we evaluated the GLUT4 content and glycogen concentration in skeletal muscle. Consistent with several previous studies (Ploug et al. 1990; Rodnick et al. 1992; Ren et al. 1994; Nakatani et al. 1997; Terada et al. 2001), the GLUT4 content in the epitrochlearis muscle was significantly higher in the Ex‐1h and Ex‐6h groups compared with the Con group after 24‐h recovery (vs. Ex‐1h: *P* = 0.023, vs. Ex‐6h: *P* = 0.020, Fig. 1A). The glycogen concentration in the epitrochlearis muscle was significantly higher in both training groups (Ex‐1h and Ex‐6h) than in the Con group after 24‐h recovery (*P* < 0.001, Fig. 1B). Furthermore, the Ex‐6h group had a significantly greater glycogen concentration compared with the Ex‐1h group after 24‐h recovery (*P* < 0.001, Fig. 1B).

### Pancreas weight, pancreas protein content and pancreatic amylase activity

**Figure 2:**
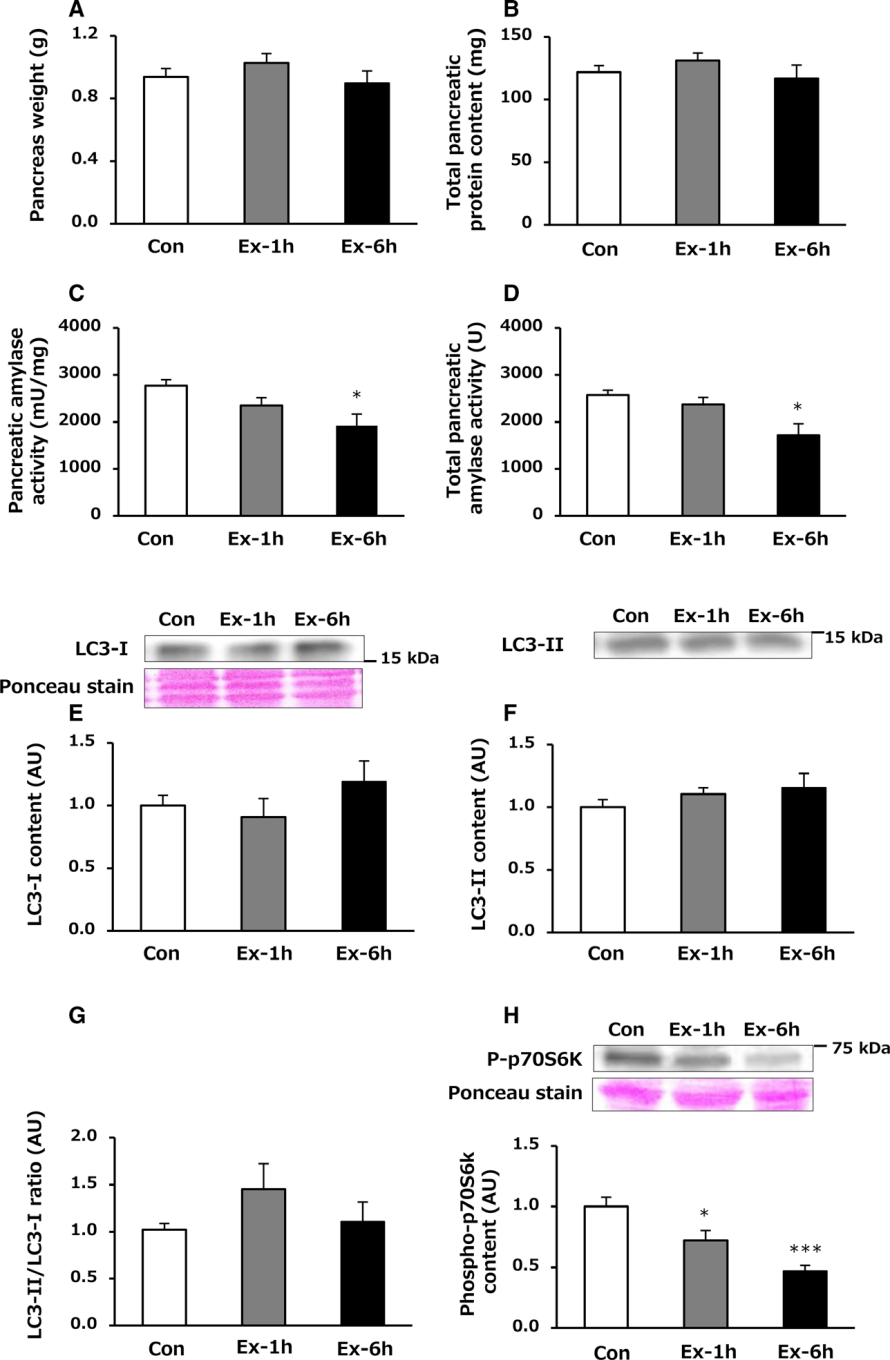
Effects of 6‐week swimming exercise training on pancreas weight (A), total pancreatic protein content (B), pancreatic amylase activity (C), total pancreatic amylase activity (D), LC3‐I content (E), LC3‐II content (F), LC3‐II/LC3‐I ratio (G), and phospho‐p70S6k content (H) in the pancreas immediately after the last exercise session. Con, control group; Ex‐1h, 1‐h swimming training group; Ex‐6h, 6‐h swimming training group; AU, arbitrary unit. Values are means ± SEM. *n* = 6–8. *P < 0.05, ***P < 0.001 vs. Con.

**Figure 3:**
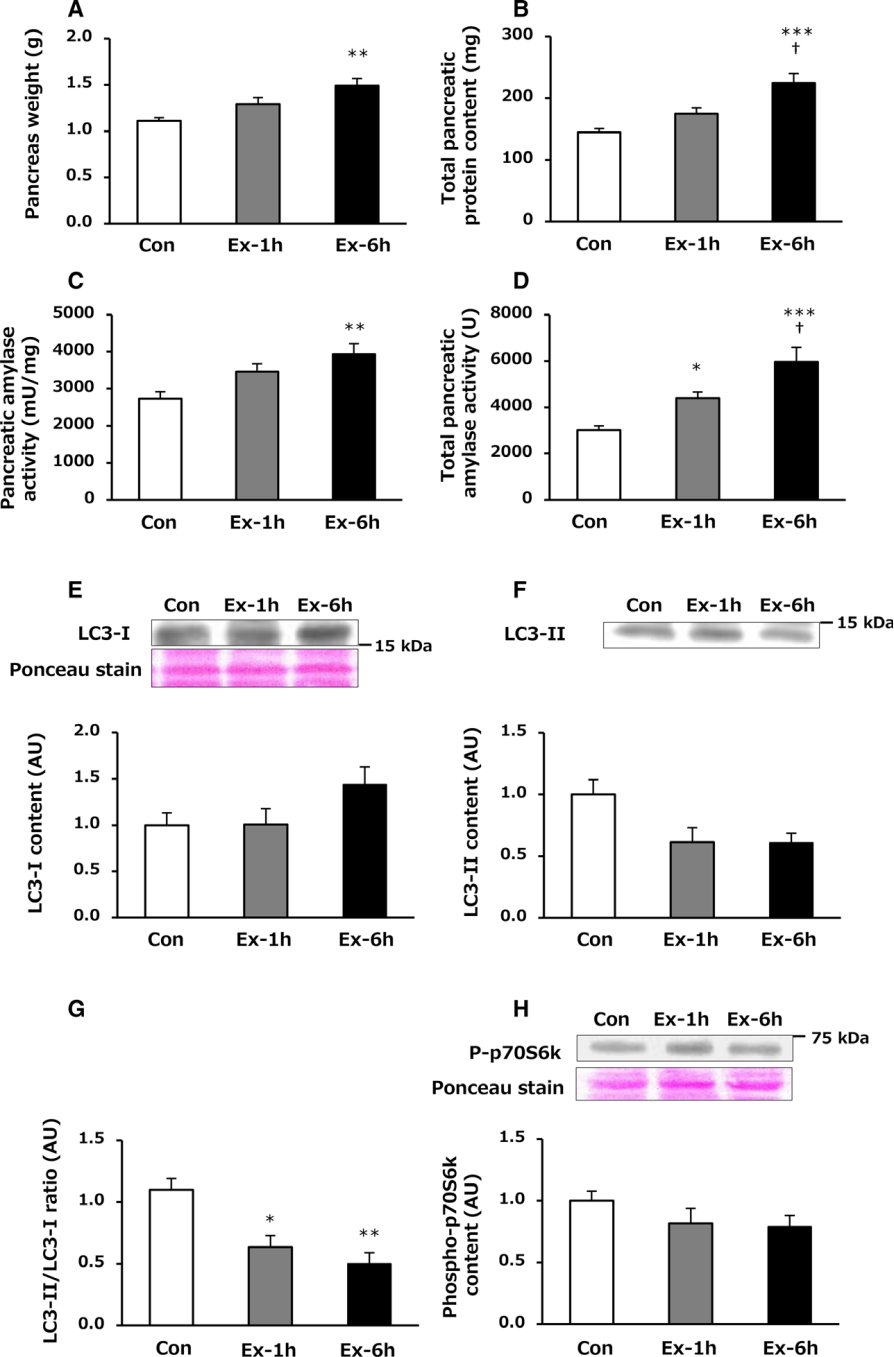
Effects of 6‐week swimming exercise training on pancreas weight (A), total pancreatic protein content (B), pancreatic amylase activity (C), total pancreatic amylase activity (D), LC3‐I content (E), LC3‐II content (F), LC3‐II/LC3‐I ratio (G) and phospho‐p70S6k content (H) in the pancreas 24 h after the last exercise session. Con, control group; Ex‐1h, 1‐h swimming training group; Ex‐6h, 6‐h swimming training group; AU, arbitrary unit. Values are means ± SEM. *n* = 6–8. *P < 0.05, **P < 0.01, ***P < 0.001 vs. Con. ^†^ *P* < 0.05 vs. Ex‐1h.

There was no significant difference in pancreas weight among the three groups immediately after the last exercise session (Fig. 2A) but the Ex‐6h group showed a significantly higher pancreas weight than that of the Con group after 24‐h recovery (*P* = 0.002, Fig. 3A).

Immediately after the last exercise session, no significant differences were found among the three groups with respect to pancreatic protein concentration (*µ*g/mg) and total pancreatic protein content (mg), which was calculated by multiplying the pancreatic protein concentration (*µ*g/mg) and pancreas weight (Fig. 2B). By contrast, pancreatic protein concentration (*µ*g/mg) in the Ex‐6h group after 24‐h recovery was significantly higher than that in the Con group (130.9 ± 5.0 vs. 150.1 ± 5.4 *µ*g/mg, *P* = 0.038). Total pancreatic protein content (mg) was significantly greater in the Ex‐6h group compared with the other two groups after 24‐h recovery (vs. Con: *P* < 0.001, vs. Ex‐1h: *P* = 0.016, Fig. 3B).

While no significant difference in amylase activity (mU/mg) was observed between the Con and Ex‐1h groups, the Ex‐6h group showed a significantly lower amylase activity (mU/mg) compared with the Con group immediately after the last exercise session (*P* = 0.013, Fig. 2C). Total pancreatic amylase activity (U), which was calculated by multiplying the pancreatic amylase activity (mU/mg) by the pancreatic weight, was not significantly different between the Con and Ex‐1h groups; however, the Ex‐6h group showed a significantly lower total pancreatic amylase activity (U) compared with the Con group immediately after the last training session (*P* = 0.010, Fig. 2D).

After 24‐h recovery, amylase activity (mU/mg) in the Ex‐6h group was significantly higher than that in the Con group (*P* = 0.007, Fig. 3C). Similarly, total pancreatic amylase activity (U) in the Ex‐1h group was significantly greater than that in the Con group (*P* = 0.046). Furthermore, the Ex‐6h group had a significantly higher total pancreatic amylase activity (U) compared with the other two groups after 24‐h recovery (vs. Con: *P* < 0.001, vs. Ex‐1h: *P* = 0.036, Fig. 3D).

### LC‐3 and p70S6k content in the pancreas

Autophagy is one of the major proteolytic systems activated in various organs in response to exercise (He et al. 2012). LC3‐II levels are associated with the formation of autophagosomes; accordingly an increased LC‐3II/LC‐3I ratio may be indicative of enhanced autophagy–lysosome activity (Mizushima & Yoshimori 2007). As shown in Figure 2, there were no significant differences among the three groups in LC3‐I content, LC3‐II content, and LC3‐II/LC3‐I ratio in the pancreas immediately after the last training session (Fig. 2E–G). However, the LC3‐II/LC3‐I ratio was significantly lower in the Ex‐1h and Ex‐6h groups compared with the Con group after 24‐h recovery (vs. Ex‐1h: *P* = 0.012, vs. Ex‐6h: *P* = 0.002, Fig. 3G).

Activation of p70S6K, a part of the signaling pathway downstream of mechanistic target of rapamycin (mTOR), is involved in the stimulation of protein synthesis (Glass 2005). Immediately after the last exercise session, pancreatic phospho‐p70S6k content in the Ex‐1h group was significantly lower than that in the Con group (*P* = 0.048, Fig. 2H). In addition, the Ex‐6h group had a significantly lower phospho‐p70S6k content compared with the Con group (*P* < 0.001, Fig. 2H). However, no significant differences were observed in phospho‐p70S6k content among the three groups after 24‐h recovery (Fig. 3H).

### Glucose transporter (GLUT2 and SGLT1) content in the small intestine (jejunum)

**Figure 4:**
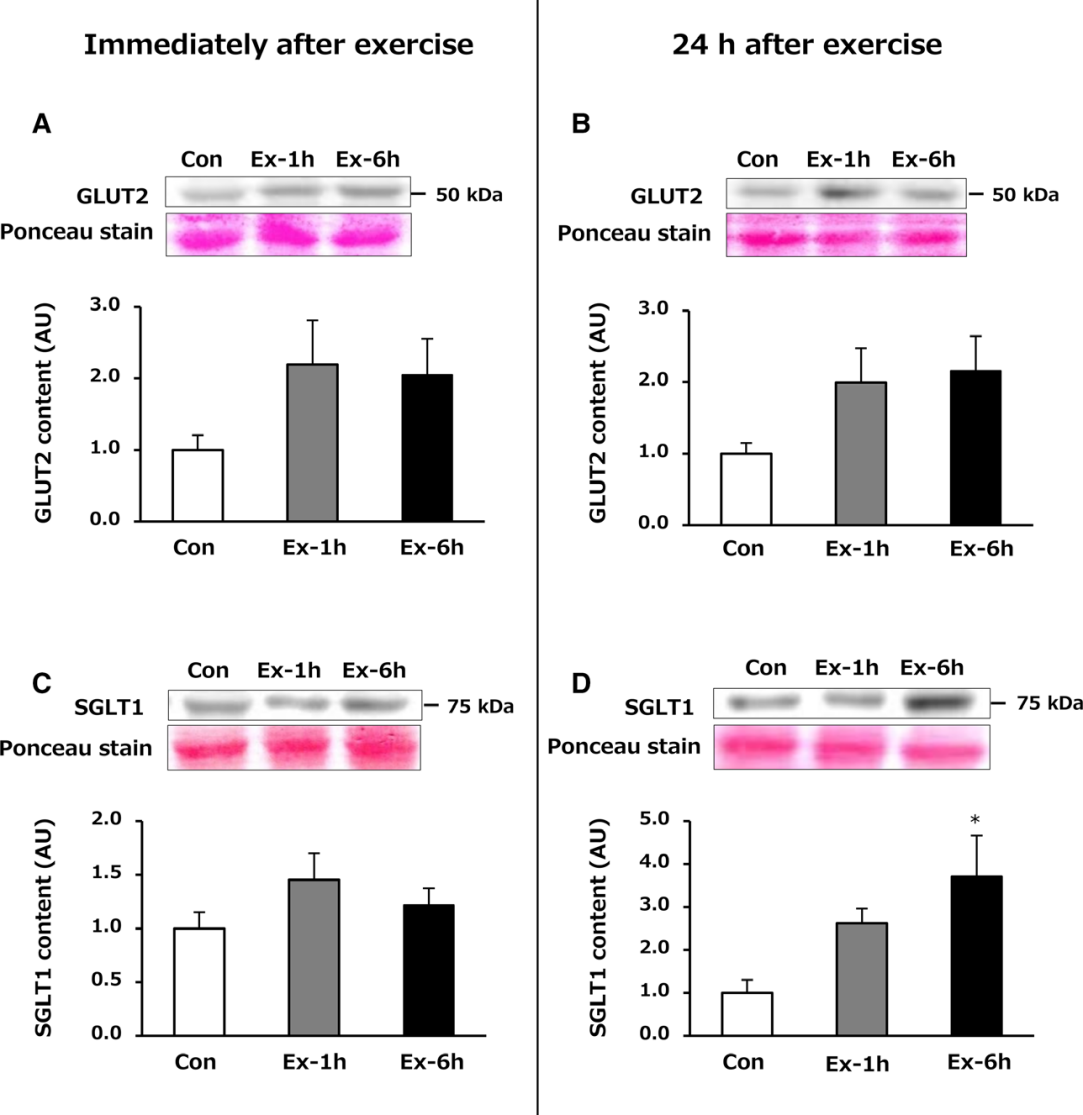
Effects of 6‐week swimming exercise training on GLUT2 (A, B) and SGLT1 (C, D) content in the jejunum. Con, control group; Ex‐1h, 1‐h swimming training group; Ex‐6h, 6‐h swimming training group; AU, arbitrary unit. Values are means ± SEM. *n* = 6–8. *P < 0.05 vs. Con.

No significant difference was observed in GLUT2 content among the three groups of rats sacrificed either immediately (Fig. 4A) or 24 h after the last exercise session (Fig. 4B). However, SGLT1 content was significantly greater in the Ex‐6h group compared with the Con group after 24‐h recovery (*P* = 0.016, Fig. 4D).

### Serum I‐FABP concentration

I‐FABP levels can be used to gauge the progression of damage in intestinal epithelial cells (van Wijck et al. 2011; van Wijck et al. 2013; Costa et al. 2017). Most of the samples were below the detection limit (20 pg/mL) immediately and 24 h after the last exercise session.

## Discussion

This study showed that long‐term endurance exercise training for a relatively longer duration (~6 h) transiently decreased total pancreatic amylase activity immediately after exercise, but also markedly increased total pancreatic amylase activity and intestinal SGLT1 protein content after 24‐h recovery. These results suggest that digestive and absorptive capacity were enhanced by this more strenuous form endurance exercise training.

Previous studies have reported that pancreatic amylase activity increased in response to 6 weeks of endurance swimming (Zsinka and Frenkl 1983) or running (Minato 1997) training for 1 h per day. Consistent with the previous studies, we found that total pancreatic amylase activity after 24‐h recovery was significantly higher in the Ex‐1h group than in the Con group (Fig. 3D), providing additional evidence that chronic exercise training for a relatively short duration (~1 h) may induce adaptation in CHO digestive capacity. In addition, total pancreatic amylase activity in the Ex‐6h group increased above that of the Control and Ex‐1h group rats after 24‐h recovery (Fig. 3D) despite decreasing immediately after the last training session (Fig. 2D). These results suggest that the longer the exercise training duration, the greater the adaptation of the pancreatic digestive capacity obtainable. As shown in Figure 3A and B, we also found that long‐term endurance exercise training for a longer duration induced substantially increases in total pancreatic protein content and pancreas weight after 24‐h recovery. Total pancreatic protein content reflects digestive enzyme content (Logsdon and Ji 2013). It thus seems plausible that other digestive enzyme levels besides amylase might be increased in the pancreas after long‐term endurance exercise training of longer duration, although we measured only pancreatic amylase activity.

To elucidate the mechanisms by which long‐term endurance training induced these adaptations in the pancreas, we evaluated signaling pathways in the pancreas involved in the synthesis and degradation protein. Whereas a previous study reported that an acute bout of exercise increased the LC3‐II/LC3‐I ratio in the pancreas (He et al. 2012), suggesting that exercise may activate the autophagic protein degradation pathway, we did not observe any changes in the LC3‐II/LC3‐I ratio immediately following exercise (Fig. 2G). The reasons for the difference in results between the previous study and our study are unclear but could be due to interspecies differences (mouse vs. rat), and/or exercise type (running vs. swimming) and so on. Instead, the Ex‐6h group had lower phospho‐p70S6k content in the pancreas compared with the Con group immediately after the last exercise session (Fig. 2H), suggesting that pancreatic protein synthesis may be suppressed after prolonged endurance exercise, resulting in lower pancreatic amylase activity, although no significant difference in total pancreatic protein content was observed. In contrast, while there was no difference in phospho‐p70S6k content among the three groups after 24‐h recovery (Fig. 3H), both training groups showed lower LC3‐II/LC3‐I ratio in the pancreas (Fig. 3G). It is therefore likely that these training‐induced increases in pancreatic amylase activity and protein content after 24‐h recovery were, at least in part, due to suppression of protein degradation. Because we were unable to measure pancreatic protein synthesis and degradation pathways throughout the 24‐h recovery, we cannot rule out the possibility that we missed the highest values of phospho‐p70S6k and LC3‐II levels during exercise and 24‐h recovery. More detailed time‐course measurements will be required in future studies.

Digested CHOs are transported across the brush border membrane by SGLT1, and passing through the basolateral membrane into the circulation via GLUT2‐mediated facilitated diffusion (Shirazi‐Beechey 1995). Because 90% of glucose absorption in the small intestine is mediated by these glucose transporters (Pencek et al. 2002), its transporter contents are likely to be a determinant factor for intestinal glucose absorptive capacity. Our results showed that the Ex‐6h group had significantly higher SGLT1 content (Fig. 4D) but not GLUT2 (Fig. 4B) after 24‐h recovery, suggesting that long‐term prolonged endurance exercise training may induce adaptations in absorptive and digestive capacities in rats. A recent study demonstrated that a 2‐week low‐carbohydrate diet substantially decreased SGLT1 content in the jejunum without GLUT2 content being altered (Higashida et al. 2019). Based on these findings, it is likely that intestinal SGLT‐1 content is more susceptible to the effects of exercise training or diets.

To obtain preliminary information on the physiological role of the adaptive increase in pancreatic amylase activity and SGLT1 content, we evaluated the relationship among variables. As shown in Table 2, strong significant correlations were observed between muscle glycogen concentrations and both total pancreatic amylase activity and SGLT1 content after the 24‐h recovery. These results suggest that increases in both digestive and absorptive capacity may be involved in the elevated muscle glycogen levels induced by chronic exercise training. It has long been believed that muscle GLUT4 content plays a key role in muscle glycogen synthesis, and our findings of a significant relationship between GLUT4 content and glycogen concentration in skeletal muscle after 24‐h recovery (Table 2) support this notion. However, in this study, the correlation coefficient between muscle glycogen concentration and total pancreatic amylase activity was significantly higher than that between the glycogen concentration and muscle GLUT4 content (*P* = 0.013). It is therefore likely that long‐term endurance exercise training increased pancreatic digestive capacity, which resulted in enhanced glucose delivery to muscle and the promoted muscle glycogen synthesis.

**Table 2:** Associations between muscle glycogen concentration and GLUT4 content, total pancreatic amylase activity and SGLT1 content after the 24‐h recovery.

	Correlation coefficient	*P* value
GLUT4 content (AU)	*r* = 0.461	*P* = 0.035
Total pancreatic amylase activity (U)	*r* = 0.868	*P* < 0.001
SGLT1 content (AU)	*r* = 0.673	*P* = 0.001

AU, arbitrary unit.

Strenuous exercise increases the supply of blood to working muscles as well as to the cardiopulmonary system in order to meet the increased demand for oxygen and nutrients while simultaneously decreasing blood flow to the gut by at least 20% (Qamar and Read 1987; van Wijck et al. 2012). This reduced flow of blood to the gut causes gastrointestinal ischemia and small intestinal injury, concomitant with an increased blood I‐FABP concentration (van Wijck et al. 2011; van Wijck et al. 2013; Costa et al. 2017). However, serum I‐FABP concentrations in most of our samples were below the detection limit immediately and 24 h after exercise. While plasma I‐FABP level was increased in response to acute strenuous exercise in some studies (van Wijck et al. 2011; van Wijck et al. 2013; Costa et al. 2017), others have reported that it was below the detection limit (Kanda et al. 2014), as in this study. In addition, as mentioned above, no deleterious effect on the glucose transporter content in the small intestine was observed after the endurance exercise training (Fig. 4). It is therefore unlikely that the swimming exercise training sessions used in this study caused ischemic damage in the small intestine in rats.

In conclusion, this study demonstrated that long‐term endurance exercise training for a longer duration induces substantial adaptations in digestive capacity in the pancreas and absorptive capacity in the small intestine in rats.

## Conflict of Interest

The authors have no conflicts of interest to disclose.
